# Supplementary material for: Horizontally transmitted symbiont populations in deep-sea mussels are genetically isolated
Source: ISME J. 2019 Aug 8;13(12):2954–68. doi: 10.1038/s41396-019-0475-z (PMC6863903; doi:10.1038/s41396-019-0475-z)
Supplement: Supplementary file 1 — Supplementary material [file 41396_2019_475_MOESM1_ESM.pdf]

Supplementary Information

SOX is the dominant community member in the Bathymodolus microbiota.

In order to investigate the community composition, we quantified the relative abundance of the microbiota species based on their genomic coverage in the metagenomes. To compare the relative abundances of the two symbionts across the different mussels, we quantified the ratio of SOX to MOX abundance, as well as the symbiont abundance relative to the mitochondrion gene abundance. For this purpose, we annotated twelve *B. brooksi* mitochondrion genes by sequence similarity to the *B. platifrons* mitochondrial genome [1] (Supplementary Fig. 2f). Our results show that SOX has a similar abundance as the mitochondrion, with a median of SOX to mitochondrion ratio of 1.06 (n=19). In contrast, MOX is much less abundant in comparison to the mitochondrion, with a median ratio of 0.0618 (n=19). The two ratios have a low variation across mussel shell sizes (Supplementary Fig. 4a,b). The comparison of SOX and MOX abundance shows that the SOX coverage is on average 19-fold higher than that of the MOX. This shows that SOX is more abundant in the mussel microbiota (Supplementary Fig. 4c). The comparison among sampling clumps shows a higher SOX to MOX ratio in sampling clump c compared to the other two (Supplementary Fig. 4c). This observation is most likely explained by differences in the availability of H_2S and CH_4 among clumps, which is a known determinant of SOX and MOX abundance in *Bathymodiolus* [2]. Thus, SOX is the dominant member in metagenomes of the mussel microbiota analysed here, where differences in the SOX to MOX ratio among the mussel metagenomes are likely determined by environmental factors.

Impact of sequencing depth on diversity analysis

We tested if the higher SNV density in SOX compared to MOX could be explained by the higher sequencing depth for SOX. To this end, we repeated part of the analyses by analyzing a subset of the orginal SOX reads that were subsampled to achieve MOX coverage levels. Normalizing SOX to the median MOX coverage of 36x resulted in a SNV density of 12.1 SNVs/kpb. This density is similar to the original estimate of 14 SNVs/kbp and still highly elevated compared to MOX (2.4 SNVs/kbp), which indicates that the difference in diversity is not driven by bias due to sequencing depth. Nucleotide diversity of SOX normalized to the MOX coverage (intra-sample π between $1.4x10^{-5}$ and $1.4x10^{-3}$, mean $4.9x10^{-4}$, ± $4.9x10^{-4}$, s.d.) results in lower estimates than for the full coverage, however, these estimates are still higher than the MOX nucleotide diversity (Table 2).

To test the effect of sequencing coverage on strain inference, we repeated the strain deconvolution for samples where the SOX coverage is decreased to the median MOX coverage. This yielded eleven SOX strains, where some are not identical to the SOX strains reconstructed from the full dataset, yet, the four SOX clades remain well supported (Supplementary Fig. 6). Hence, we consider the strain relationships as more reliable indicators of strain diversity than the number of strains alone. Nevertheless, the resulting AFS does not reveal modes according to the strain clades as observed previously in the full coverage analyses (Supplementary Fig. 7).

Individual-specific microbiota genetic variants cannot be explained by host genetics.

Mitochondrial genes have been previously used to identify lineages in *Bathymodiolus* mussels [3]. Here, we used genetic variants in the mitochondrion genome as a marker to investigate the contribution of mussel relatedness to the observed genetic isolation of the symbiont communities. Analysing the twelve mitochondrial genes, we detected 175 SNVs with a density of 15.6 SNVs/kbp (intra-sample $\pi^{Mitochondria}$ between $9.5x10^{-6}$ and $7.7x10^{-5}$, mean $3.3x10^{-5} \pm 2.2x10^{-5}$, s.d.). We found that most of the mitochondrial SNVs are fixed (frequency≥0.95) with an average of 99.8% fixed SNVs per sample. The phylogeny of the dominant mitochondrial haplotype for each sample shows no clustering of individuals according to sample clump or mussel size (Supplementary Fig. 10a), which indicates that the mussel individuals analyzed here belong to the same population. The high proportion of fixed SNVs results in high F_{ST} values (Supplementary Fig. 10b,c), which is expected for vertically transmitted mitochondrial genomes. We further used the F_{ST} values to detect associations between mussel genetics and symbiont diversity. Our results reveal no association between mussel F_{ST} and symbiont F_{ST} for any of the two symbionts (Supplementary Fig. 10b,c). Consequently, we conclude that the population structure observed for SOX and MOX cannot be explained by mussel relatedness or location.

Strain symbiont composition based on ribosomal proteins

Ribosomal proteins are frequently used for analyzing bacterial diversity. Here we studied the phylogenetic relationships among the reconstructed strains by analyzing ten different ribosomal protein-coding genes present in the core genomes of the two symbiotic species. The phylogenetic tree reconstruction shows that both species clearly split in the tree (Supplementary Fig. 5). However, the low intra-species bootstrap values indicate that relationships among strains can not be confidentially inferred. This can be traced back to the low number of variants within SOX and MOX, respectively. This results in poorly resolved phylogenetic networks for the SOX and MOX

populations. In conclusion, because ribosomal proteins are highly conserved, using them for the analysis of within-species diversity leads to the underestimation of the existing diversity.

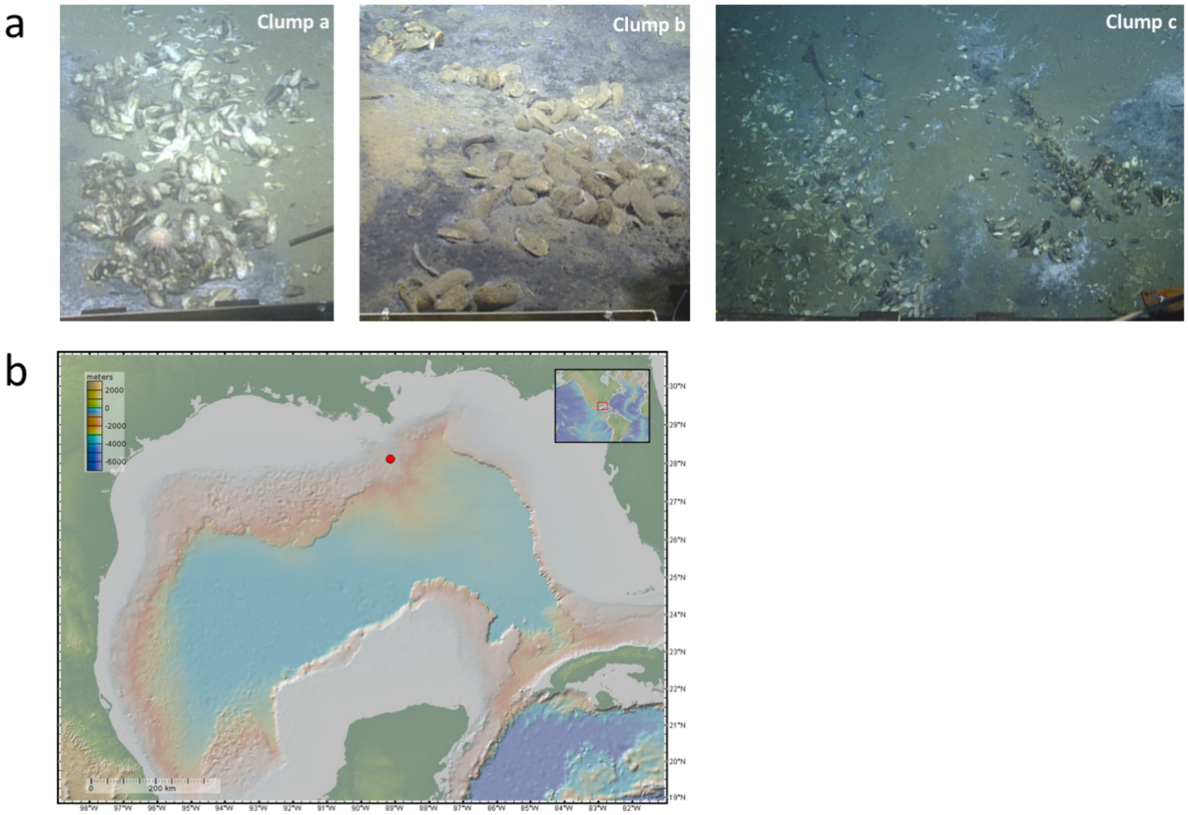

Figure S1: Sampling information. a, sampling clumps. **b,** sampling location at the Gulf of Mexico, different color shades represent sampling depth in meters.

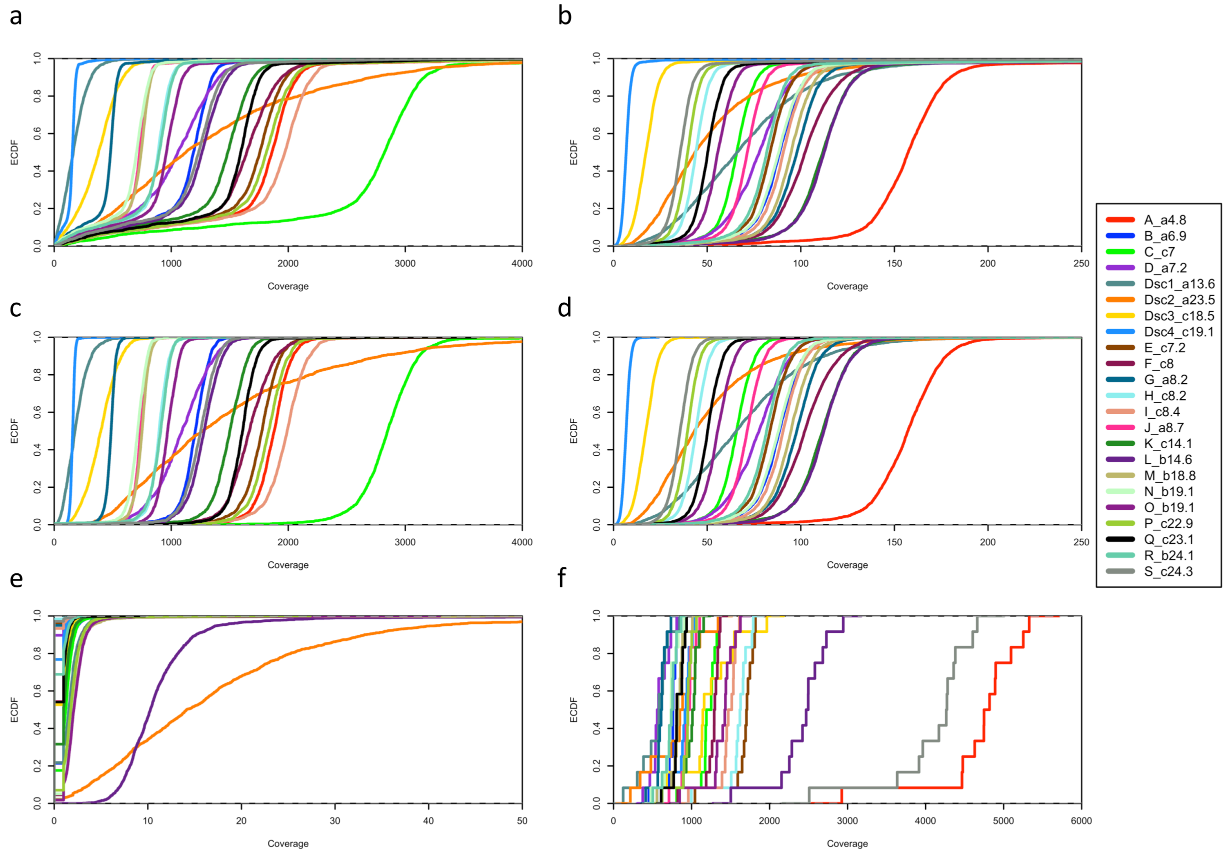

Figure S2: Coverage of symbiont bins. Empirical cumulative distribution function (ECDF) of MAGs and mitochondrion. Samples are additionally labeled with the location where they have been sampled and the shell size. 19 samples (A-R) are used for the analysis of population structure and the remaining 4 samples (Dsc1-Dsc4) were discarded during the analysis; Dsc1 and Dsc2 were discarded before the binning due to high variance in symbiont marker gene coverages (Dsc1_a13.6: median coverage=217.41 and SD=98.14 for SOX marker genes, median coverage=70.47 and SD= 41.3 for MOX marker genes; Dsc2_a23.5: median coverage=1368.28 and SD=785.73 for SOX marker genes, median coverage=44.60 and SD=185.12 for MOX marker genes). Dsc3 and Dsc4 were discarded after binning due to low coverage (median coverage < 15x for MOX core genes and median coverage <350x for SOX core genes). Median and standard deviation (SD) across genomes were estimated using only non-discarded samples. **a,** SOX incl. outlier genes (1 910 genes, median coverage range: 482-2 822, SD range: 157-891), **b,** MOX incl. outlier genes (2 618 genes, median coverage range: 36-157, SD range: 97-298), **c,** SOX (1 439 genes, median coverage range: 486-2 849, SD range: 52-294), **d,** MOX (2 518 genes, median coverage range: 36-157, SD range: 6-21), **e,** MGS3 (1 449 genes, median coverage range: 0-10, SD range: 0-11), no outlier genes detected. Total genome length of 1.31 Mbp. The third MGS could not be assigned to a taxonomic level; the top three genera found are Oceanicella (Taxonomy ID:1233054) with 61 genes (4.21%), Neomegalonema (Taxonomy ID 356797, 26 genes, 1.79%), and Micavibrio (Taxonomy ID 213485, 26 genes, 1.79%). This unknown species is present in very low abundance and was mainly found in sample O. **f,** Mitochondrion (median coverage range: 568-4 784; SD range: 78-618).

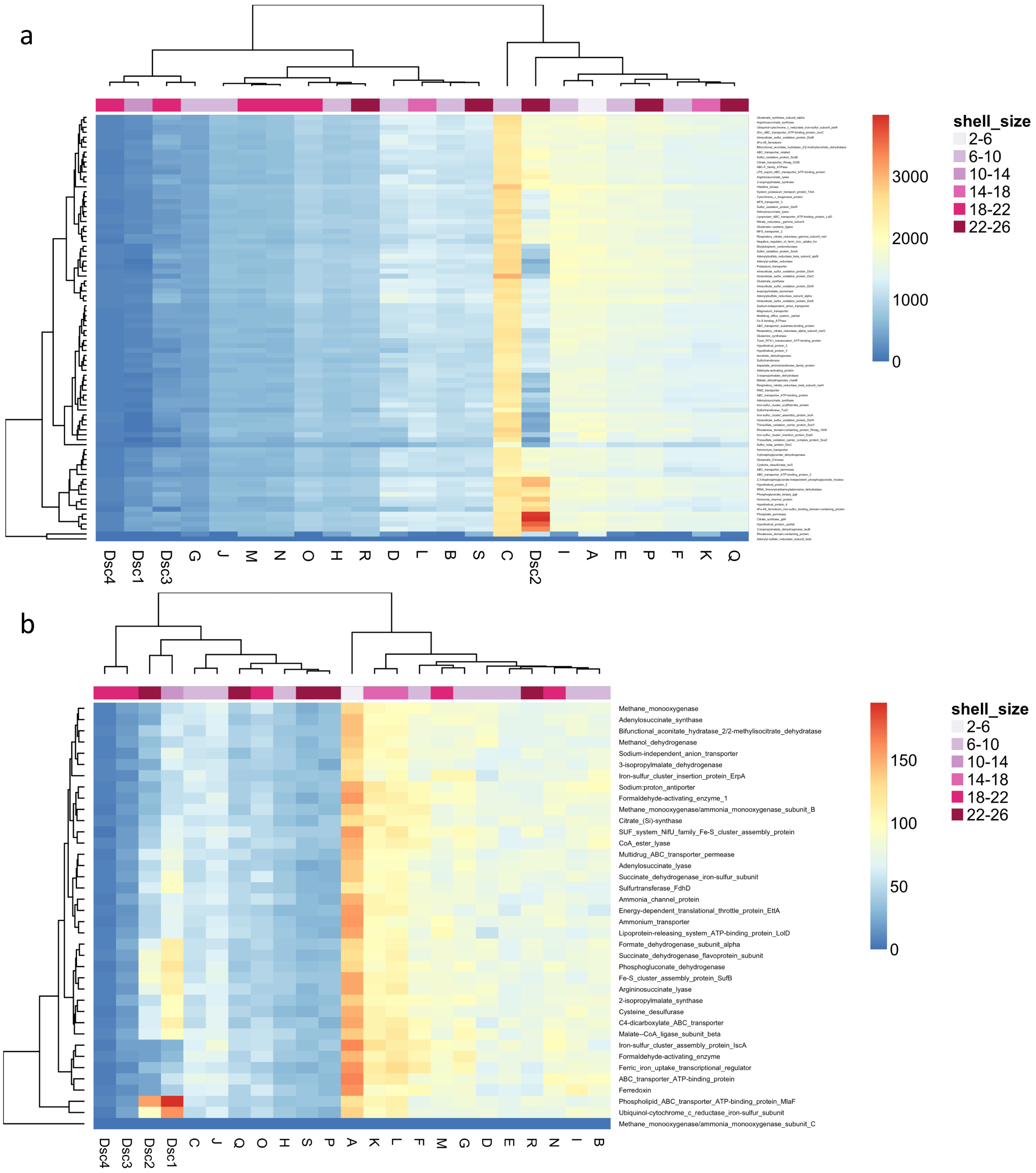

Figure S3: Gene coverages for a, 86 SOX and b, 39 MOX marker genes. The two core genomes were validated by screening for the presence of known SOX and MOX marker genes [4]. These genes encode for protein functions in the methane and sulfur/thiosulfate metabolism pathways, such as methane monooxygensase (PmoB), methanol dehydrogenase (XoxF), intracellular sulfur-oxidation proteins (Dsr), sulfur-oxidation proteins (Sox), and adenylsulfate reductase (Apr), as well as for proteins associated to the carbon metabolism, such as malate, succinate and formate dehydrogenases (Mdh, Sdh, and Fdh). A total of 39 MOX and 86 SOX marker genes are present in the gene catalog.

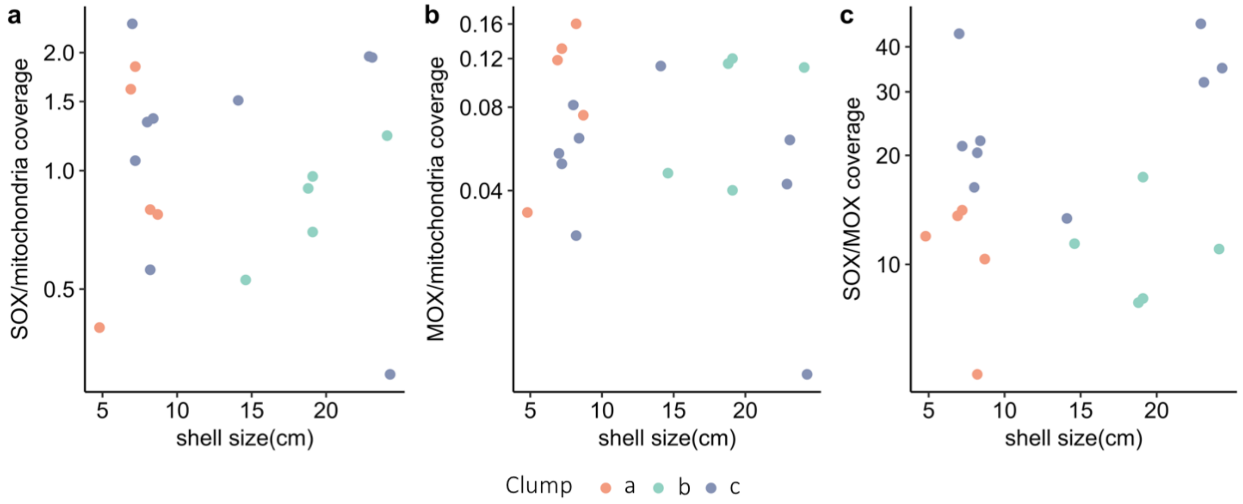

Figure S4: Relative symbiont abundances across mussels. Note that the y-axis (ratio) is displayed in log scale. Coverages are estimated by calculating the mean coverage across all genes for SOX, MOX, and mitochondria, respectively.

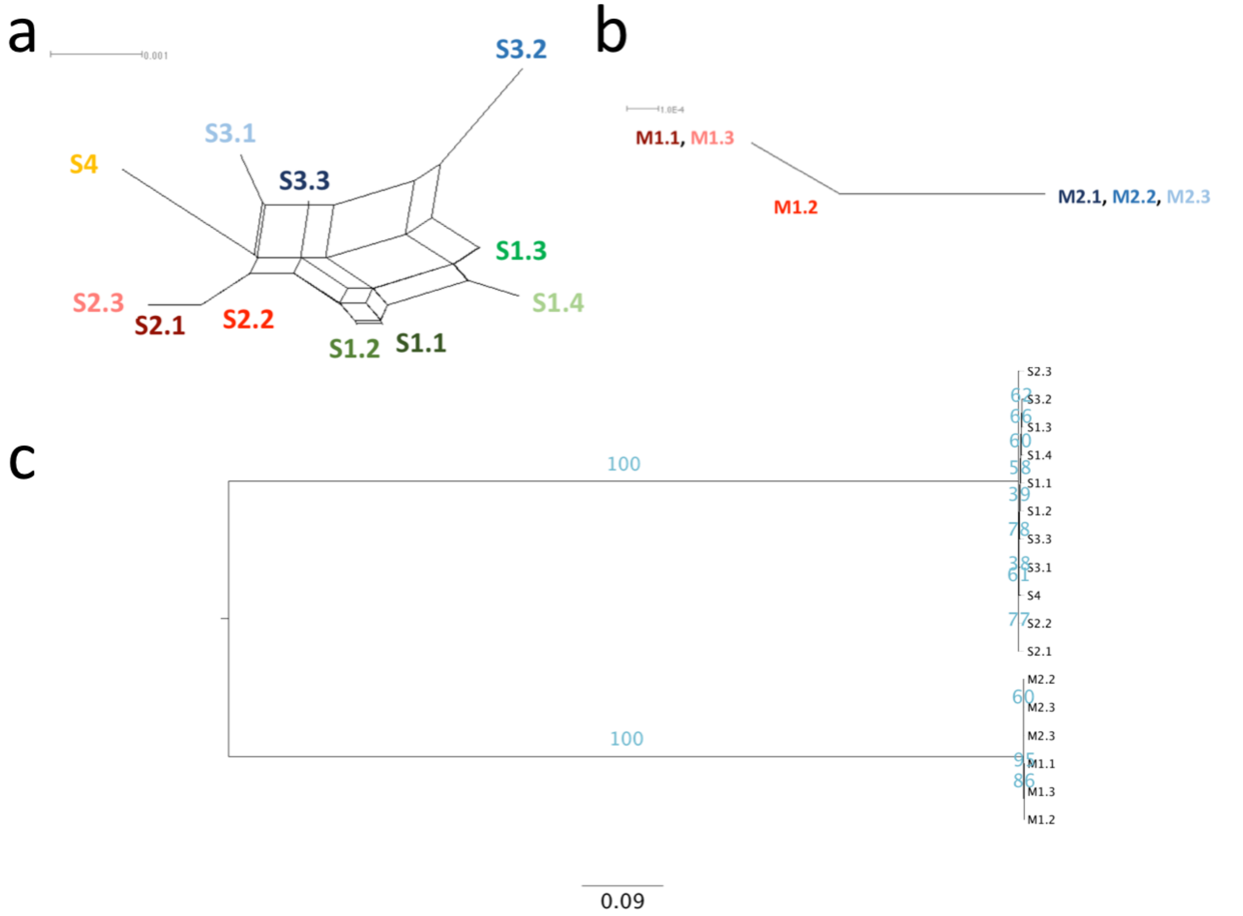

Figure S5: Splits networks and phylogenetic tree showing strain relationships based on a sequence alignment of ten ribosomal protein-coding genes. a, SOX splits network. The number of variant sites in the alignment is 26 out of 3 489 (0.7%), where nine sites are parsimony informative. Six variants are located at the first codon position and 20 variants are located at the third codon position. **b,** MOX splits network. The number of variant sites in the alignment is 3 out of 3 147 (0.1%), where none is parsimony informative. One variant is located at each codon position. **c,** Maximum likelihood phylogenetic tree reconstructed from the concatenated SOX and MOX merged alignment. The branch labels represent bootstrap values. The scale indicates the number of substitutions per site. The inferred length of the branch splitting SOX and MOX species ancestors is 1.75 substitutions per site.

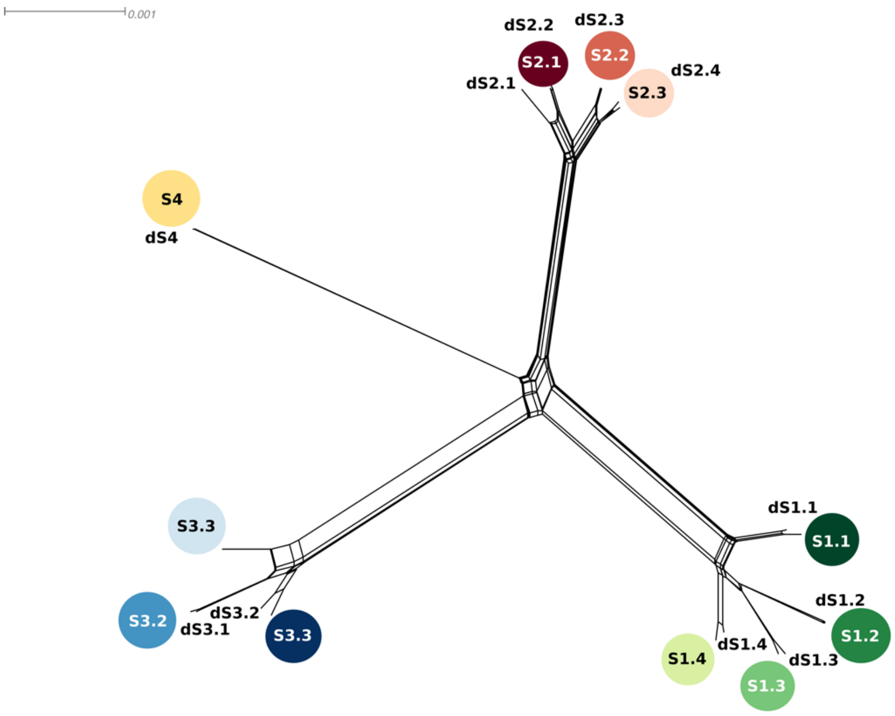

Figure S6: Splits network for SOX strains reconstructed using the full data set (colored) and SOX strains reconstructed after normalizing SOX to the median MOX coverage (black).

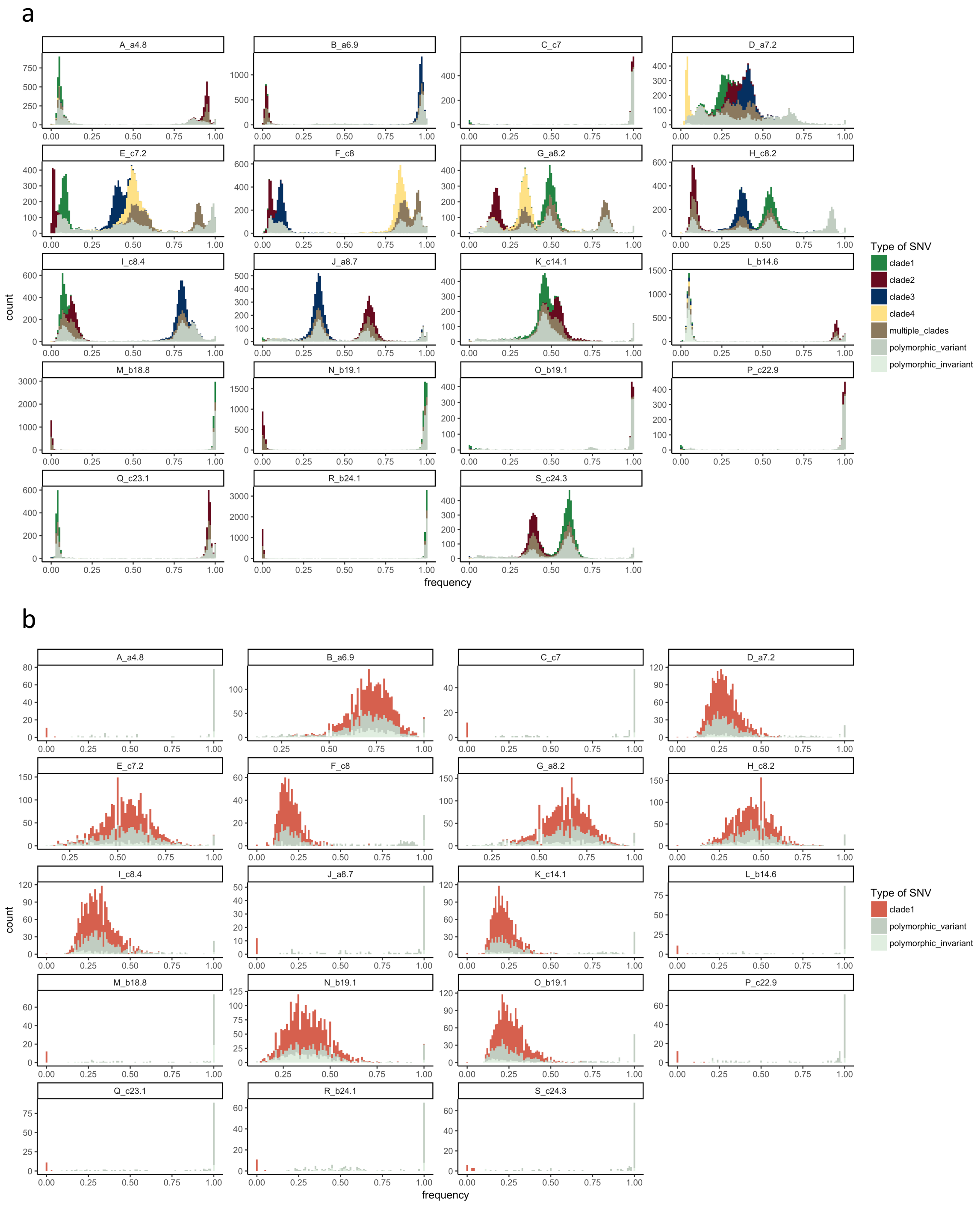

Figure S7: Intra-sample unfolded allele frequency spectra for a, SOX and b, MOX. Stacked histogram of the frequencies colored by strain clades (see colors in Fig. 1). SNVs are defined as clade-specific when they occur at the same state in every strain from a clade, as variant, if they differ among strains from one clade and invariant if they are not recovered by the strain deconvolution.

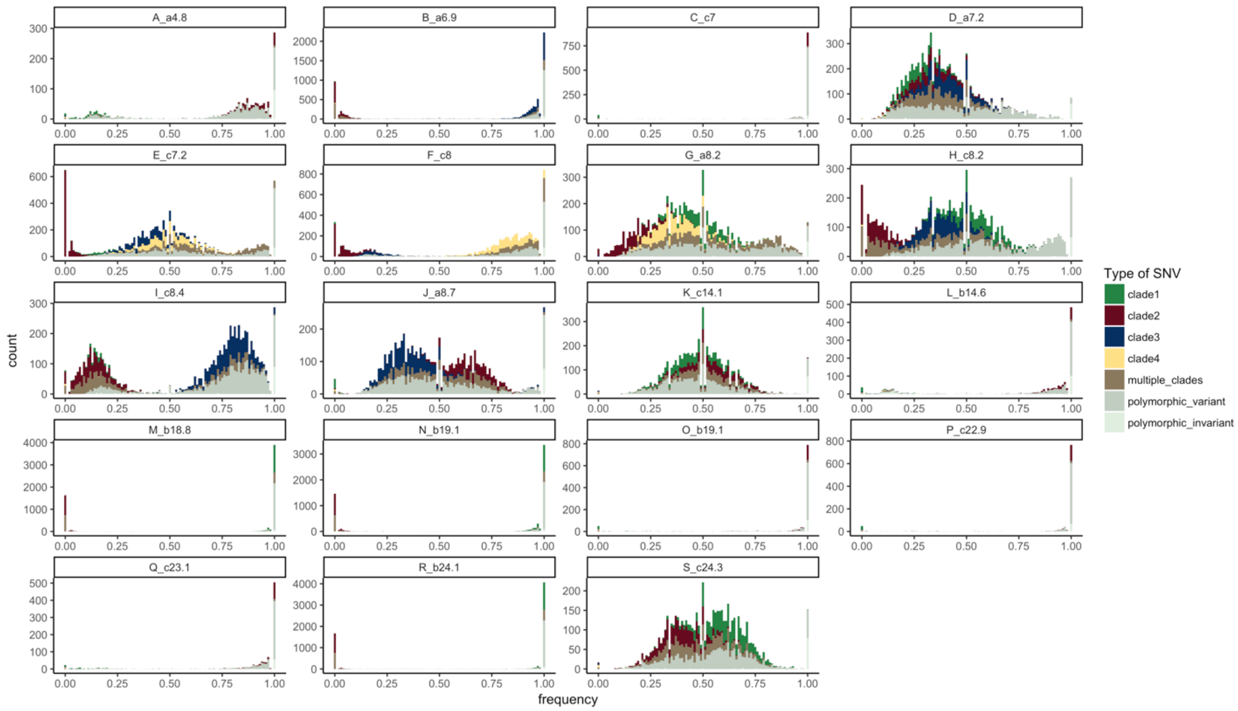

Figure S8: Intra-sample unfolded allele frequency spectra for downsampled SOX to MOX coverage (~36x). See also legend in Supplementary Fig. 7.

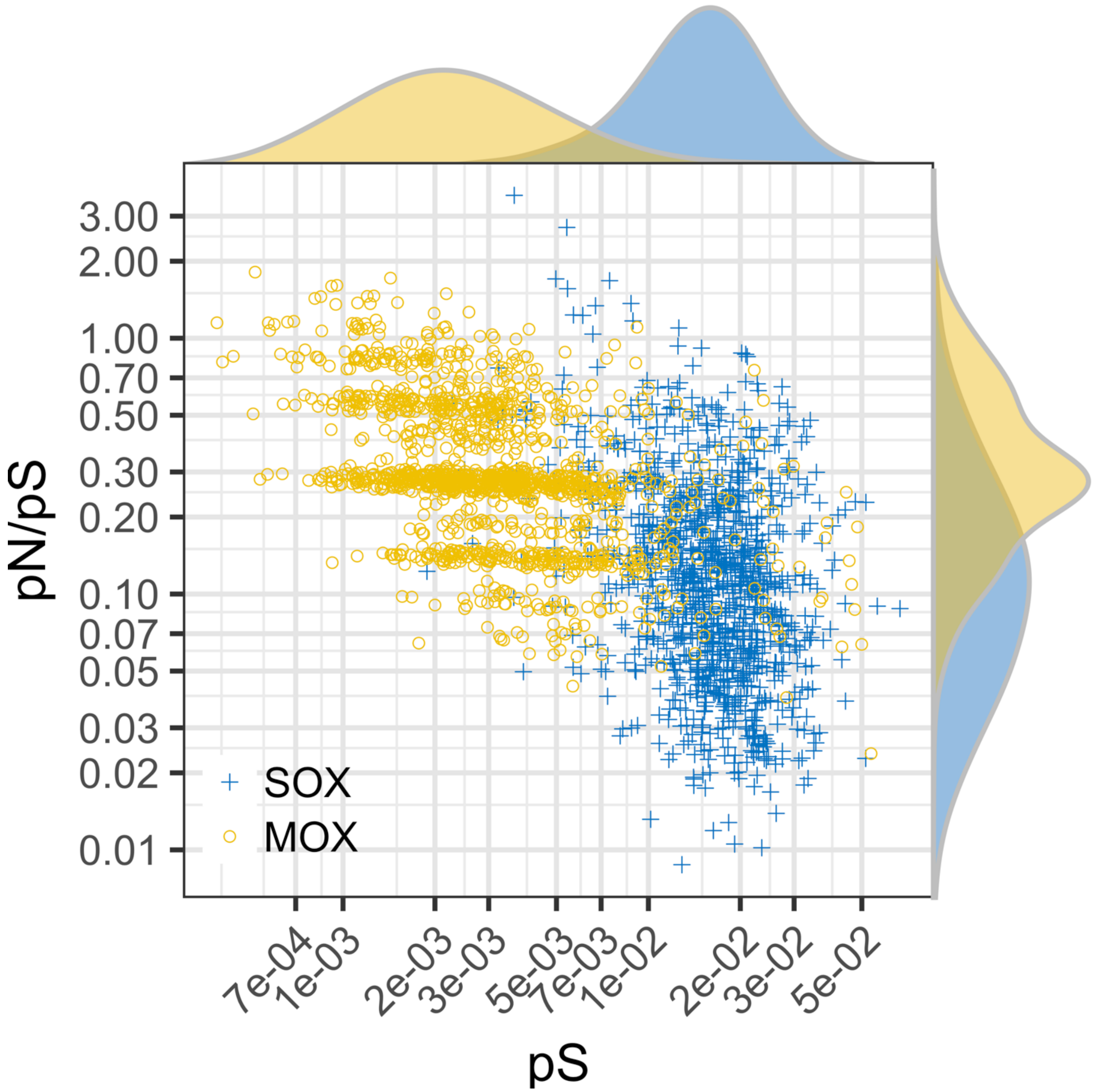

Figure S9: pS and pN/pS across genes for both symbionts. Density plots of the distributions are given in the margins. Note the log scale of both axes. Median MOX pS (0.0029) is smaller than median SOX pS (0.015) (Wilcoxon rank sum test, p-value < 10^{-6}). Median MOX pN/pS (0.28) is larger than median SOX pN/pS (0.12) (Wilcoxon rank sum test, p-value < 10^{-6}.). Only genes with SNVs are considered (1 117 genes for SOX and 1 359 genes for MOX).

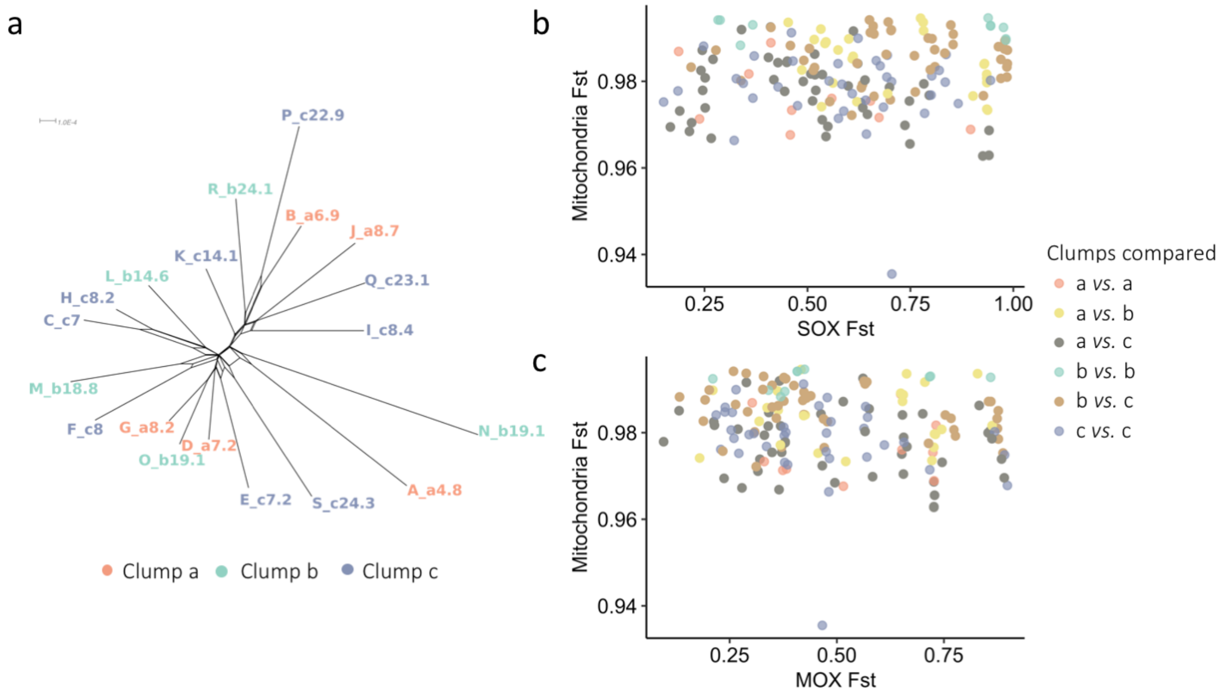

Figure S10: Mitochondria diversity. a, splits network [5] (using Uncorrected P distances) based on the dominant haplotypes. We retrieved dominant mitochondrial haplotypes for each of the mussels by identifying SNVs with alternative frequency >0.5 and assigning the alternative nucleotide to these positions. **b,c,** relationship between **b**, SOX and **c**, MOX F_{ST} and mitochondria F_{ST}.

Table S1: Report of raw and processed sequencing data and assembly statistics for the 23 mussel metagenomes from the northern Gulf of Mexico. #Polymorphic SNVs gives the number of SNVs with a frequency between 0.05 and 0.95. Sample-specific SNVs is the number of SNVs that are present in only one sample and are not detected in any other sample. #Differential dominant strain SNVs gives the number of SNVs that are specific for dominant haplotypes and are retrieved from samples that have a dominant strain (strain frequency ≥90%). We retrieved dominant haplotypes for the mussels with a dominant strain by identifying SNVs with alternative frequency ≥0.9 and assigning the alternative nucleotide to these positions.

Sample	Size (cm)	Clump	# Reads	# Trimmed reads	# Contigs	# Genes	# Non-redundant genes	# Polymorphic SNVs SOX	# Polymorhic SNVs MOX	# Polymorhic SNVs mitochondria	# Sample-specific SNVs SOX	# Sample-specific SNVs MOX	Dominant strain (≥90%)	#Differential dominant strain SNVs
A_a4.8	4.8	a	37,061,240	37,028,936	1,394,249	1,044,714	697,354	3740	30	3	10	6	-	-
B_a6.9	6.9	a	37,133,252	37,106,306	1,486,937	1,116,948	688,707	1852	3026	1	140	169	-	-
C_c7	7	c	38,285,976	38,252,301	1,418,404	1,006,084	693,453	207	27	0	17	2	S2.1	-
D_a7.2	7.2	a	36,755,366	36,731,976	1,560,929	1,129,326	683,199	11064	2138	0	20	7	-	-
E_c7.2	7.2	c	37,933,850	37,904,916	1,498,588	1,091,911	706,758	10505	2831	1	13	43	-	-
F_c8	8	c	38,060,325	38,033,705	1,432,041	1,074,170	697,580	8923	826	0	242	2	-	-
G_a8.2	8.2	a	37,230,553	37,203,081	1,453,270	1,133,968	679,642	10404	2934	0	22	73	-	-
H_c8.2	8.2	c	38,070,443	38,034,179	1,548,988	1,150,117	699,488	10812	2717	0	28	51	-	-
I_c8.4	8.4	c	38,134,036	38,103,876	1,470,797	1,067,621	706,089	10148	2232	0	52	7	-	-
J_a8.7	8.7	a	37,807,348	37,782,857	1,463,875	1,138,457	678,274	7147	48	0	49	2	-	-
K_c14.1	14.1	c	36,693,086	36,661,232	1,460,859	1,084,916	698,127	6742	1542	0	24	6	-	-
L_b14.6	14.6	b	37,412,053	37,386,973	1,459,725	1,106,129	687,149	3338	39	0	1903	3	-	-
M_b18.8	18.8	b	39,118,344	39,086,779	1,781,668	1,033,575	693,534	314	40	0	14	12	S1.4	42
N_b19.1	19.1	b	37,259,078	37,231,365	1,479,982	1,141,813	691,418	397	2536	0	18	398	S1.4	74
O_b19.1	19.1	b	38,172,030	38,137,180	1,505,470	1,132,309	707,064	315	1934	0	16	26	-	-
P_c22.9	22.9	c	39,623,187	39,589,960	1,473,410	1,101,708	693,062	162	48	0	10	3	S2.2	44
Q_c23.1	23.1	c	38,172,522	38,140,344	1,457,910	1,097,379	683,899	615	43	2	12	3	S2.2	44
R_b24.1	24.1	b	37,435,085	37,410,384	1,492,285	1,134,884	689,875	341	89	0	37	44	S1.4	45
S_c24.3	24.3	c	38,814,311	38,775,353	1,549,186	1,151,312	705,170	6423	32	1	31	5	-	-
Dsc1_a13.6	13.6	a	33,794,815	33,711,379	1,599,186	1,058,588	585,047	-	-	-	-	-	-	-
Dsc2_a23.5	23.5	a	39,994,621	39,945,569	1,285,996	862,788	402,105	-	-	-	-	-	-	-
Dsc3_c18.5	18.5	c	38,976,397	38,944,379	1,734,610	1,211,676	670,826	-	-	-	-	-	-	-
Dsc4_c19.1	19.1	c	36,555,894	36,518,986	1,604,292	1,176,485	703,808	-	-	-	-	-	-	-

References

1. Sun J, Zhang Y, Xu T, Zhang Y, Mu H, Zhang Y, et al. Adaptation to deep-sea chemosynthetic environments as revealed by mussel genomes. *Nat Ecol Evol* 2017; **1**: 0121.
2. Riou V, Halary S, Duperron S, Bouillon S, Elskens M, Bettencourt R, et al. Influence of CH_4 and H_2S availability on symbiont distribution, carbon assimilation and transfer in the dual symbiotic vent mussel *Bathymodiolus azoricus*. *Biogeosciences* 2008; **5**: 1681–1691.
3. Breusing C, Vrijenhoek RC, Reusch TBH. Widespread introgression in deep-sea hydrothermal vent mussels. *BMC Evol Biol* 2017; **17**.
4. Ponnudurai R, Kleiner M, Sayavedra L, Petersen JM, Moche M, Otto A, et al. Metabolic and physiological interdependencies in the *Bathymodiolus azoricus* symbiosis. *ISME J* 2017; **11**: 463–477.
5. Huson DH. SplitsTree: analyzing and visualizing evolutionary data. *Bioinformatics* 1998; **14**: 68–73.
